# Consistency of biological networks inferred from microarray and sequencing data

**DOI:** 10.1186/s12859-016-1136-0

**Published:** 2016-06-24

**Authors:** Veronica Vinciotti, Ernst C. Wit, Rick Jansen, Eco J. C. N. de Geus, Brenda W. J. H. Penninx, Dorret I. Boomsma, Peter A. C. ’t Hoen

**Affiliations:** Department of Mathematics, Brunel University London, London, UK; Johann Bernoulli Institute of Mathematics and Computer Science, University of Groningen, Groningen, The Netherlands; VU University Medical Center, Amsterdam, The Netherlands; Leiden University Medical Center, Leiden University, Leiden, The Netherlands

**Keywords:** Gaussian graphical models, Gene regulatory network, Microarray, Next-generation sequencing

## Abstract

**Background:**

Sparse Gaussian graphical models are popular for inferring biological networks, such as gene regulatory networks. In this paper, we investigate the consistency of these models across different data platforms, such as microarray and next generation sequencing, on the basis of a rich dataset containing samples that are profiled under both techniques as well as a large set of independent samples.

**Results:**

Our analysis shows that individual node variances can have a remarkable effect on the connectivity of the resulting network. Their inconsistency across platforms and the fact that the variability level of a node may not be linked to its regulatory role mean that, failing to scale the data prior to the network analysis, leads to networks that are not reproducible across different platforms and that may be misleading. Moreover, we show how the reproducibility of networks across different platforms is significantly higher if networks are summarised in terms of enrichment amongst functional groups of interest, such as pathways, rather than at the level of individual edges.

**Conclusions:**

Careful pre-processing of transcriptional data and summaries of networks beyond individual edges can improve the consistency of network inference across platforms. However, caution is needed at this stage in the (over)interpretation of gene regulatory networks inferred from biological data.

**Electronic supplementary material:**

The online version of this article (doi:10.1186/s12859-016-1136-0) contains supplementary material, which is available to authorized users.

## Background

One important direction in systems biology is to discover gene regulatory networks from transcriptional data based on the observed mRNA levels of a large number of genes. The nodes of the network are genes and the edges are the corresponding interactions, such as activation, repression or translation. Transcriptional data can be generated using two different high-throughput technologies: gene expression microarrays [18] and tag-based sequencing methods, like DeepSAGE [12, 21] and RNA-seq [19].

Statistical models have been proposed in the literature for reverse engineering networks from data and different adaptations have been developed to deal with the high dimensionality and complexity of biological networks in particular, e.g. [8, 15, 22, 31]. Amongst these approaches, Gaussian graphical models have shown to be particularly popular. The computationally efficient method introduced by [8] allowed the estimation of these models for the case of a large number of nodes relative to the sample size (*p*≫*n*) via the use of an *L*_1_ penalised likelihood approach. This approach is suited to microarray data, as the data are continuous and, after normalization, well-approximated by a multivariate normal distribution. A number of papers have extended the original model to different cases, such as dynamic networks from microarray data [1], hub-type networks from microarray data [31], condition-specific networks from microarray data [7] and networks from next generation sequencing data, which are discrete, e.g. [4, 36].

After the advent of next generation sequencing technologies, a number of studies have evaluated the consistency between the two platforms, both at the level of expression values and at the level of differentially expressed genes, e.g. [12, 27, 30, 33, 37]. The general conclusion from these studies is that sequencing technologies not only allow to identify transcripts that have not been previously annotated, but they also allow to better quantify very low and very high expression transcripts, which would be masked by microarray’s background noise and saturation effects, respectively. In the intermediate range, there is high replication and detection amongst the two platforms, although platform specific and dataset-specific effects can limit the level of consistency significantly [27]. A small number of studies has gone beyond expression and differential expression. In particular, [29] studied the consistency of clustering methods on microarray and RNA-seq data and [11] studied the consistency of co-expression networks on microarray and RNA-seq data, where the networks are inferred by Pearson correlation values.

Linked to the work of [11], the aim of this paper is to quantify the consistency, across platforms and samples, of biological networks inferred by sparse Gaussian graphical models. We consider a rich dataset containing samples that are profiled under both microarray and sequencing techniques as well as a large set of independent samples [39]. We assess the consistency of networks both at the level of individual edges and at the level of enrichment among pathways extracted from the Kyoto Encyclopedia of Genes and Genomes (KEGG) database (http://www.genome.jp/kegg). For the latter, we make use of a recently developed test for network enrichment [28].

## Method

### Data

The data used in this study contain DeepSAGE (DS) sequencing of 21bp tags and corresponding Affymetrix expression data from total blood RNA samples from unrelated individuals from the Netherlands Twin Register (NTR) [5] and the Netherlands Study of Depression and Anxiety (NESDA) [24]. From the NTR/NESDA cohorts, we selected healthy (and thus non-diabetic) individuals at the extremes of the fasting glucose serum level distribution: 41 individuals with fasting glucose concentrations ≤ 4.8 mmol/l; 53 individuals with fasting glucose concentrations ≥ 5.9 mmol/l. This selection comprised 28 males and 66 female individuals. Microarray and DeepSAGE data generation, processing and quality control have been described previously [13, 35, 39]. In addition, we used Affymetrix-profiled blood samples of 1272 additional participants of the NTR and NESDA studies, selected using the same glucose based criterion as above. In particular, of these there are 418 high glucose and 854 low glucose samples. We later refer to the three datasets as DS (the 94 DeepSAGE samples), MA(DS) (the 94 corresponding microarray samples) and MA(Add) (the 1272 additional microarray samples). Together with gene expression data, a number of corresponding covariates are used: age (in years), sex, Body Mass Index (BMI), glucose level and smoking (yes and no). These were obtained during the interview at the time of blood draw. Glucose was measured in blood plasma using the Vitros 250 glucose assay (Johnson and Johnson).The DS samples are corrected for GC content.

**Fig. 1:**
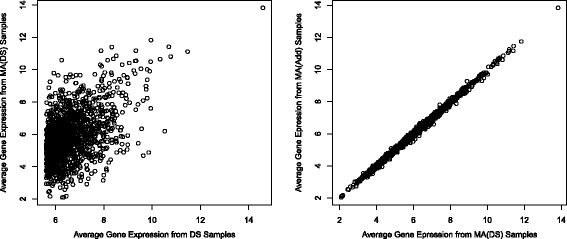
DS versus microarray expression. *Left*: Average (log) expression for the 1435 genes from the 94 DS samples (x-axis) and the 94 microarray samples (y-axis). *Right*: Average gene expression from the 94 microarray samples versus the 1272 additional microarray samples

For the analysis, we select the 1500 most highly expressed genes for which there are concept profiles, i.e. for which there is information in the literature in at least 5 papers. This group of genes is expected to be least affected by observational noise in their expression measurements and, therefore, to be most consistent across platforms. This aids in focussing on the actual contribution of network modelling to the consistency across platforms, which is the focus of this paper. From these 1500 genes, we select 1435 genes that are common to both DS and microarray data. For microarray data, we take the average expression of all probes targeting the same gene. Figure 1 (left) shows the correspondence between count data and expression data for the 1435 genes, averaged over the 94 samples. The correlation between the two is 0.49, suggesting a moderate reproducibility across the two platforms at the level of expression data. The right plot shows a very high reproducibility for the microarray experiments between the 94 samples and the 1272 independent samples.

### Sparse Gaussian graphical models

In this paper, we use Gaussian graphical models for inferring networks from data. A Gaussian graphical model makes the assumption that the vector of nodes *D* follows a multivariate Gaussian distribution, so

$$D \sim N(\mu,\Sigma), $$

with mean vector *μ* and variance-covariance matrix *Σ*. Of particular importance is the inverse of the variance-covariance matrix, also called precision or concentration matrix, which is usually denoted by

$$\Theta= (\theta_{ij})=\Sigma^{-1}. $$

This matrix holds a special role in Gaussian graphical models: in fact, zeros in the precision matrix correspond to conditional independence between the corresponding variables, i.e. the absence of an edge in the corresponding graph. In particular, there is a direct link between the precision value *θ*_*ij*_ and the partial correlation *ρ*_*ij*_ between *D*_*i*_ and *D*_*j*_ conditioning on all other nodes, as

1 $$ \rho_{ij}=-\frac{\theta_{ij}}{\sqrt{\theta_{ii}\theta_{jj}}}.   $$

Thus inferring the network of interactions can be re-casted into the problem of estimating the precision matrix *Θ* and extracting its zero structure. Of particular importance for the analysis in this paper is the fact that the diagonal of the matrix *Θ* is given by the inverse of the conditional variances, i.e. $\theta _{ii} = \frac {1}{{\text {var}}(D_i|D_{j},j\ne i)}$ [34]. Thus, the scale of individual nodes can play a significant role in the dependency structure.

In the case of high-dimensional networks, that is where the sample size *n* (number of experiments) is smaller than the number of nodes *p* (number of genes), a sparse estimate of the precision matrix *Θ* can be obtained by imposing an *L*_1_-penalty constraint on the entries of the precision matrix. This results in the penalised likelihood optimization

$$\max_{\Theta}\left[ \log |\Theta|-\text{Trace}(S\Theta) -\lambda||\Theta||_{1} \right], $$

with *S* the sample covariance matrix and *λ* the penalty parameter controlling sparsity. [8] provide an efficient optimization procedure for this problem, by maximising the penalised log-likelihood iteratively for each node and, at each step, by re-writing the problem into an equivalent lasso regression problem. The latter is estimated efficiently using coordinate descent methods.

### Network inference

We adopt a Poisson regression model for the DeepSAGE data to correct for spurious confounders in measuring the interaction between the genes. Let *Y*_*i*_=(*Y*_*i*1_,…,*Y*_*ip*_) be the count data for gene *i* under *p* experiments. Let *X*=(*X*_1_,…,*X*_*c*_) be a vector of covariates. Then

$$\begin{array}{@{}rcl@{}} Y_{ij} &\sim & {\text{Poisson}}(\lambda_{ij}) \\ \log(\lambda_{ij}) &=&\log(n_{j})+\sum_{c=1}^{C} x_{jc}^{T}\beta_{ic}, \end{array} $$

with *n*_*j*_ the total number of counts in experiment *j*, *x*_*j*_=(*x*_*j*1_,…,*x*_*jC*_) the vector of covariates for sample (experiment) *j* and *β*_*i*_ the vector of parameters for gene *i*. For microarray data, a multiple regression model is used to correct for the same covariates, with the exception of GC content and total number of counts which are specific to count data.

We then extract the residuals of the regression models. For the Poisson regression, we take the deviance residuals defined by

$$d_{ij}=\text{sign}(y_{ij}-\hat\lambda_{ij})\sqrt{2y_{ij} \log \frac{y_{ij}}{\hat\lambda_{ij}}-2(y_{ij}-\hat\lambda_{ij})}. $$

These are approximately normally distributed [20] and are used for network modelling.

This two-step method does not take into account the uncertainty of the regression estimates and could, especially when the number of samples is similar to the number of regressors, lead to biased estimates. We account for this uncertainty by non-parametrically bootstrapping the data and repeating the analyses on the bootstrap samples. This provides typically asymmetric confidence intervals of the quantities of interest that will account both for the bias and the under-estimated variance of the original two-step estimation procedure.

In order to assess the impact of individual node variances and of correction for confounding effects on the resulting inferred network and on the consistency of network models across different samples and platforms, we fit sparse Gaussian graphical models in the following three cases:

1. Residuals standardised to have mean zero and variance one per node.
2. Residuals not standardised.
3. Normalised expression data standardised to have mean zero and variance one but not corrected for confounding effects.

For the first and the third case, we use the package huge [38], which automatically scales the data prior to network inference. In terms of the choice of the penalty parameter *λ*, we select this based on the rotation information criterion (ric) approach, which is available in the R function huge.select. We take the optimal network for the case of standardised residuals from the 94 DS samples. This returns a network with 1435 nodes and 29865 edges. We then select *λ* for all other networks in such a way that all networks in the comparative study are of similar size. For the second case, we use the function glasso in the package glasso [9], which does not automatically scale the data.

Given the estimated networks, the test developed by [28], and implemented in the R package neat, is used to detect enrichment of the networks among KEGG pathways. In particular, the test detects whether the number of edges between two pathways in the inferred network is larger than what is expected by chance. For this, we download all human KEGG pathways using the R package KEGGREST [32]. Out of the total 299 pathways, we filter 62 pathways as those that contain at least 20 of the selected genes and test for enrichment amongst any pair of pathways. Finally, we rank the *p*-values and build a network with 62 nodes (the pathways) and with edges corresponding to the top enrichments.

Throughout the analysis, the agreement between any two networks is measured using the product-moment correlation between the corresponding adjacency matrices. This is implemented in the function gcor of the R package sna. The function qaptest in the same package is used to compute the *p*-values under a re-labelling of the nodes of the network.

## Results and discussion

### The confounders effect

In a first set of experiments, we evaluate the impact of confounders on network inference and thus justify the choice of performing the network modelling on the residuals. In order to do this, we fit networks under two cases. In the first case the data are scaled but not corrected for confounders (with the exception of GC and number of experiments for DS data). In the second case, the data are scaled and corrected for confounders as explained before.

**Fig. 2:**
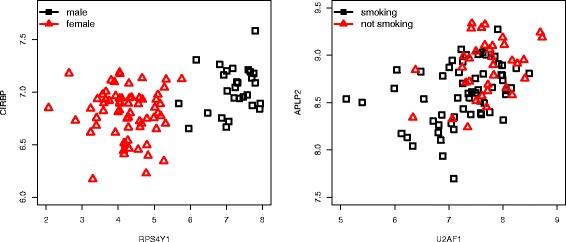
Confounders effect. Two examples of the effect of confounders on the MA(DS) network: the two links are found when not correcting for confounders, but not after correction

The results on our data show a high correlation between the networks in the two cases, with 95 % bootstrapped confidence intervals (0.56,0.94) for DS, (0.68,0.75) for MA(DS) and (0.95,0.98) for MA(Add). The agreement is particularly high in the MA(Add) case due to the larger sample size. However, looking at the difference between the two networks for each of the three datasets, we can see how genuine regulatory interactions, when one transcript directly regulates the expression of another transcript, may be masked by confounding effects. Figure 2 shows two examples of edges that are found in the MA(DS) network when not correcting for confounders but they are not found when correcting for confounders. In general, any two differentially expressed genes may be highly correlated, but they may not be directly interacting, i.e. this may be a spurious correlation caused by a third factor. One way of distinguishing between direct and indirect interactions is by correcting for confounders: if the correlation is still at the the level of residuals (i.e. partial correlation), then it may be a sign of a genuine relationship.

In conclusion, regulatory interactions between genes may be masked by confounders effects. Although their effect in the network reconstruction is found to be small for our particularly study, performing this step increases the chances of detecting genuine regulatory mechanisms. For the remaining of the paper, we therefore fit networks to the residuals, after correcting for the confounders mentioned in the description of the data.

### The node variance effect

The fact that the variance of a node has an impact on the dependency structure is natural for models that are based on estimating the inverse of covariances, as explained in the description of Gaussian graphical models. Due to computational stability of the estimation procedure, in most cases the variables are standardized prior to the estimation of the dependency structure. However, this is not always included in the implementations that are made available. For example, the original implementation of sparse Gaussian graphical models in the glasso package [9] does not automatically standardize the variables. Of 44 citations of the package in Google scholar, we found that 14 use glasso for inferring biological networks, and only 3 of these make explicit mentioning to standardization of the data. This is the same for JGL [6], where the variables are only centralised per condition, and for SparseTSCGM [2], where the variables are not standardized. Amongst other implementations of sparse Gaussian graphical models, huge [38] automatically scales the data, and similarly, the function sugm in the flare R package [16] is based on estimation of the inverse of the correlation matrix and, thus, is scale independent. These are only few examples of the most popular implementations. In general, the decision as to whether to scale the data or not is not always done automatically by the software, so it is important to appreciate the impact of this choice on the resulting network and the implications when interpreting the network for biological findings.

**Fig. 3:**
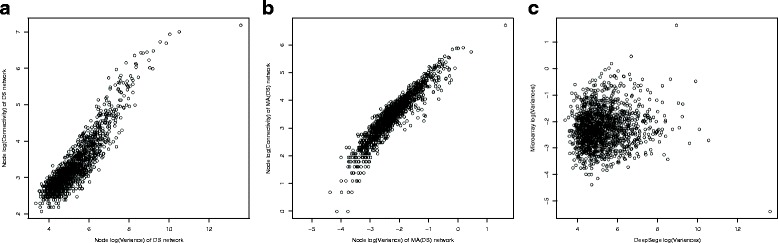
Node variance effect. Node connectivity versus node variance for DS network (**a**), MA(DS) network (**b**) and node variance from DS data versus node variance from MA data (**c**)

Figure 3 plots the connectivity of each node versus its variance (both in the log scale) for the networks inferred from non-scaled data (case 2). Figure 3 (a) is for the case of DS data, whereas (b) is for the case of MA(DS) data. A similar relationship exists for the MA(Add) data. The plots show how the connectivity of a node is strongly linked with its variance. The panel (c) of the figure shows how the variance of a node is not consistent across platforms. Thus the conclusion is that the networks inferred in this analysis from non-scaled data will mainly reflect measurement scale and platform specific effects rather than biological effects.

**Fig. 4:**
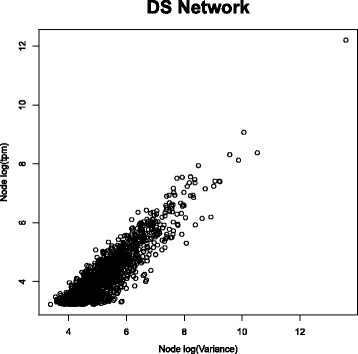
Node Connectivity versus Expression. Node connectivity of DS network versus node expression level (measured as number of transcripts per million (tpm))

In addition, Fig. 4 shows how the residuals with the largest variances tend to correspond to the highly expressed genes. Looking at the list of these genes, we find various markers for cellular composition. In particular, as the data come from blood samples, many of the highly expressed genes are related to blood markers, e.g. HBB is the gene with the highest variance and is the most connected gene of the DS network (1307 edges), whereas HLA-C is the highest connected gene in the MA(DS) network (811 edges). Markers for cellular composition are in general not expected to have also a regulatory role, thus the network on non-scaled data may show features that, in some cases, may be consistent across platform but they may not necessarily be linked to regulation.

**Fig. 5:**
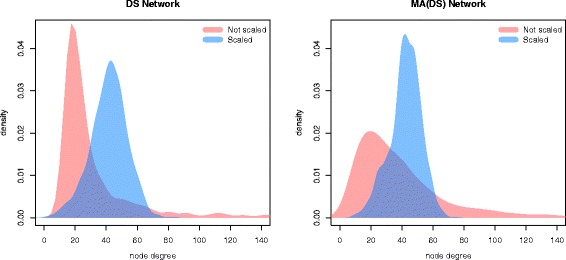
Scaling Effect on Node Connectivity. Node degree distributions of DS (*left*) and MA(DS) (*right*) networks on scaled (*red*) and non-scaled (*blue*) data. The networks have similar size (about 30000 edges)

In general, the connectivity of a network inferred from non-scaled data is strongly influenced by the individual node variances. As shown by Fig. 5, the network on non-scaled data has a very pronounced right tail, i.e. a small number of highly connected nodes (hubs), whereas the network on scaled data has a more uniform level of connectivity. The plots show how the effect is more pronounced for the DS than for the MA(DS) network, as in count data the variance scales with the mean and there is therefore a larger variability in node variances.

If networks on non-scaled data exhibit a gene variance effect and if the measurement scales are not consistent across platforms, then one would expect a lower consistency of networks across samples and platforms if the data are not standardized. Table 1 shows the correlations of networks across different samples and platforms, distinguishing the case of scaled and not-scaled data. The correlation between adjacency matrices is computed using the function gcor of the R package sna.

**Table 1:** Correlation among the 6 networks from expression data (DS, MA(DS) and MA(Add)) and two cases (SCALED - data centered to mean zero and variance one for each gene - and NOT SCALED)

		DS		MA(DS)		MA(Add)	
		SCALED	NOT SCALED	SCALED	NOT SCALED	SCALED	NOT SCALED
DS	SCALED	1.00	0.18	0.04	0.02	0.06	0.05
	NOT SCALED		1.00	0.03	0.03	0.04	0.04
MA(DS)	SCALED			1.00	0.36	0.26	0.21
	NOT SCALED				1.00	0.14	0.22
MA(Add)	SCALED					1.00	0.54

Firstly, the table shows varying levels of correlations, which all tested significant using the qaptest function (*p*-values <0.001). Secondly, the networks on the same data, but scaled versus non-scaled, are rather different, particularly for the DS case, where the correlation is only 0.18. This is less pronounced for the MA(Add) case, due to the larger sample size. Thirdly, the correlation across samples improves when the data are scaled, e.g. 0.26 between MA(DS) and MA(Add) when they are both scaled versus 0.22 when they are not scaled, and 0.06 between DS and MA(Add) when they are both scaled versus 0.04 when they are not. The correlations between the scaled networks tested significantly larger than those between the non-scaled networks (*p*-values <0.001). Fourthly, the correlation across platforms is significant, but generally very low (top second and third quadrant), even when the data are scaled. We will expand on this point in the next section.

### Agreement of enrichment networks

**Fig. 6:**
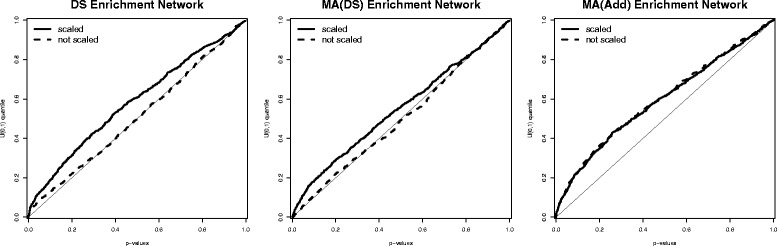
Enrichment of Links between Pathways. q-q plot of *p*-values of the enrichment test for all pairwise comparisons of 62 KEGG pathways for DS, MA(DS) and MA(Add) and distinguishing the case of scaled and not-scaled data

Table 1 shows a very small agreement of network models, particularly across different platforms. The question could therefore be asked whether the overlap between the two networks is at all biologically relevant. In this section, we aim to summarise the networks at the higher level of functional groups and interactions between these. In particular, we summarise the networks in terms of interactions among 62 KEGG pathways. The test neat [28] is used to detect enrichment among any pair of pathways. Figure 6 shows the quantile-quantile plots (q-q plots) of the *p*-values for all pairwise comparisons. Under no enrichment, the *p*-values should follow a uniform distribution. In that case, the q-q plot would follow the diagonal line. For the case of DS and MA(DS), it is obvious how scaling the data returns networks that are enriched of biological edges, as the q-q plots are those of right-skewed distributions. The node variance effect of the networks on non-scaled data may therefore mask biological facts and the detection of biologically meaningful interactions. For the case of MA(Add), there is detection of interactions among pathways both for the networks on scaled and non-scaled data. In fact, Table 1 showed a relatively large agreement between the two networks (correlation 0.54). This is most likely due to the significantly larger sample size of MA(Add) (1272 versus 94), which limits the effect of the variances of individual nodes on the network inference.

**Fig. 7:**
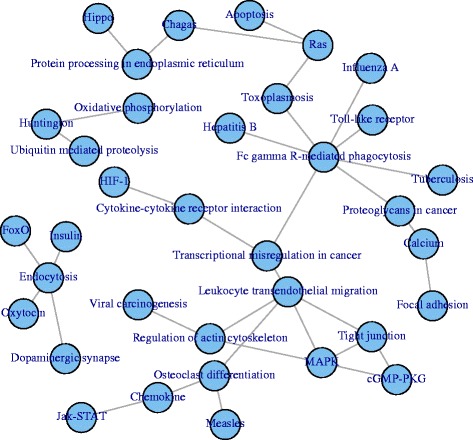
Network of pathways overlap. Overlap of Pathway Networks from DS, MA(DS) and MA(Add) at 10 % significance level

Considering the case of scaled data, we build networks among pathways testing for "Overenrichment" at a 10 % significance level. The resulting networks have 240 edges in the case of DS, 240 edges for MA(DS) and 427 edges for MA(Add). Figure 7 shows the intersection of the three networks. The network reveals some links between pathways that are supported by existing literature. For example, the link between the Focal Adhesion and Calcium pathways is found significant in the DS network (*p*-value 0.006, 34 links between the two pathways), MA(DS) (*p*-value 0.041, 32 links) and MA(Add) (*p*-value 0.009, 39 links). Looking closely at the links, there are many connections between the protein tyrosine kinase 2 (PTK2B) from the calcium pathway with genes in the focal adhesion pathway, for example a link between VAV1 and PTK2B in the DS network that was found previously by [10]. In the other direction, AKT2 from the focal adhesion pathway was found to be regulated by calcium signalling [26] and the link between AKT2 and calcium-dependent regulators such as CALM3, which is found in the microarray networks, is supported by [23, 25].

Table 2 shows the agreement among the three networks in terms of correlation. Comparing this table with Table 1, we observe the same agreement between MA(DS) and MA(Add) (*p*-value 0.532), but a significantly higher agreement across platforms: 0.11 versus 0.04 for DS-MA(DS) (*p*-value 0.019) and 0.12 versus 0.06 for DS-MA(Add) (*p*-value 0.017). Overall, this suggests a higher level of consistency at the level of interactions between pathways, rather than at the level of individual edges.

**Table 2:** Correlation among the networks at the level of KEGG pathways

	DS	MA(DS)	MA(Add)
DS	1.00	0.11	0.12
MA(DS)		1.00	0.26
MA(Add)			1.00

**Fig. 8:**
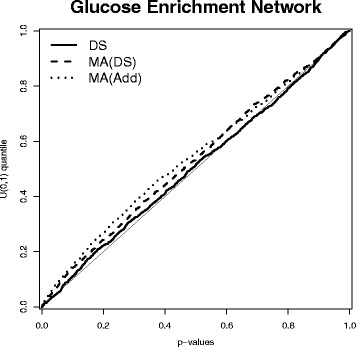
High versus Low Glucose Networks. q-q plot of the enrichment test for all pairwise comparisons of 62 KEGG pathways for the differential networks between high and low glucose

In many cases, the biological objective of the analysis is to detect differences in regulatory patterns among biological conditions. Then the interest is in the differential networks, that is in the edges that are found only in one of the conditions. Consistency of differential network analyses among different samples and platforms is therefore also important. In order to assess this, we fitted networks on high glucose and low glucose samples separately. A similar agreement to that in Table 1 was found across platforms, both for high and low glucose networks. We then considered the networks containing the edges that are in high glucose but not in low glucose. We found 18686 edges unique to high glucose from the networks inferred from DS data, 25522 edges in the networks inferred from MA(DS) data and 15974 edges in the networks inferred from MA(Add) data. But the three networks altogether have only 100 edges in common, suggesting that the detection of differences at the level of individual edges is not robust. In contrast to this, when enrichment among pathways is considered, Fig. 8 shows a low level of pathway enrichment for all three networks, particularly for the network from the DS data. Similar results are obtained when considering the networks unique to low glucose. For example, there are 21218 edges unique to high glucose from the networks inferred from DS data, 24684 edges in the networks inferred from MA(DS) data and 13489 edges in the networks inferred from MA(Add) data, but the three networks altogether have only 98 edges in common. This means that the networks, across samples and platforms, have little signature of differences between high and low glucose conditions. Of course, there may be genuine differences, but there is not enough evidence in the data to pick these up. These examples show that consistency across platforms can be particularly low for differential networks, since one is looking for a robust detection of edges that are in one condition but not in the other condition, so sensitivity as well as specificity of sparse Gaussian graphical models play a role in this case.

## Discussion and conclusion

The aim of this paper was to assess the consistency of networks inferred by sparse Gaussian graphical models across different samples and data platforms. To this aim, we used a rich dataset containing samples that are profiled under both techniques as well as a large set of independent samples. We first of all showed the impact of confounding effects (such as age and gender) on the network reconstruction. The effect was not very strong in our study. Nevertheless, we show how confounding effects may return spurious interactions amongst genes and may mask the search for genuine regulatory interactions. Although the inference method does not correspond to any generative model of the data, i.e., it is impossible to set up a sampling scheme that exactly correspond to the two-step inference procedure, we have investigated how realistic sampling schemes for genetic networks are affected by confounding variables. The results, included in the Additional file 1, show that the inferred precision matrix in the two-step procedure relates closely the underlying network in all kind of confounding scenarios. Moreover, [3] show that the precision matrix can approximately be interpreted in terms of conditional odds ratios, which are more natural ways to interpret conditional independence for count data. Given these considerations, we recommend to devise an appropriate regression model and fit networks to the residuals of this model, i.e. to data adjusted for confounders.

Our analysis of the inferred networks shows that individual node variances can have a remarkable effect on the connectivity of the resulting network. In particular, they result in hub-type networks with hubs made of the nodes with the highest variances. The inconsistency of node variances across platforms and the fact that the variability level of a node may not be linked to its regulatory role mean that, failing to scale the data prior to the network analysis, leads to networks that are not reproducible across different platforms and that may be misleading. This point is of particular importance given that not all available implementations of sparse Gaussian graphical models automatically scale the data and thus this step is often left to the user. Failure to scale the data prior to network modelling may in part explain the belief, particularly in the early days of network modelling of biological systems, that biological networks are scale-free and the later contributions which questioned this assumption, e.g. [14, 17] and references therein.

However, even after scaling of the data, our analysis shows that a large number of edges are not replicated across platforms. We then show how the reproducibility of networks across different samples and platforms is notably higher if networks are summarised in terms of enrichment amongst functional groups of interest, such as KEGG pathways, rather than at the level of individual edges. In particular, we show, for the case of differential networks, how conclusions from individual edges are not consistent across platforms and, once again, how conclusions drawn from analyses of individual edges may be misleading.

Overall, while the field of network modelling makes steady advances and new network models with higher specificity, sensitivity and computational efficiency are proposed in the literature, this study shows that caution is needed at this stage in the (over)interpretation of the inferred networks for biological findings. In particular, we show how summarising the networks at the level of functional groups of interest, such as KEGG pathways, provides a more robust representation of the underlying network and allows to reach conclusions that are most consistent across platforms. The network of functional groups is also of a significantly smaller scale than the network of genes and, thus, it can be more easily interrogated to generate hypotheses that can be tested by further biological experiments.

## Abbreviations

BMI, Body Mass Index; DS, DeepSAGE; KEGG, Kyoto encyclopedia of genes and genomes; MA, MicroArray; NESDA, Netherlands study of depression and anxiety; NTR, Netherlands twin register; q-q plot, quantile-quantile plot; SAGE, serial analysis of gene expression

## Additional file

Additional file 1 Simulation showing the effect of confounders on network reconstruction. (PDF 117 kb)
