# Supplementary material for: Consistency of biological networks inferred from microarray and sequencing data
Source: BMC Bioinformatics. 2016 Jun 24;17:254. doi: 10.1186/s12859-016-1136-0 (PMC4919861; doi:10.1186/s12859-016-1136-0)
Supplement: Additional file 1 — Simulation showing the effect of confounders on network reconstruction. (PDF 117 kb) [file 12859_2016_1136_MOESM1_ESM.pdf]

METHODOLOGY ARTICLE

Supplementary Materials for Consistency of biological networks inferred from microarray and sequencing data

Veronica Vinciotti, Ernst C. Wit, Rick Jansen, Eco J.C.N. de Geus, Brenda W.J.H. Penninx, Dorret I. Boomsma and Peter A.C. 't Hoen

Full list of author information is available at the end of the article

Confounder effect on network reconstruction

In order to study the effect of sequentially removing confounders on the subsequent network reconstruction, we performed a simulation study. We considered 4 different scenarios, in which 2 genes, Y_1 and Y_2, interact in the presence of a single confounder X. In each of the four simulations, the confounder X_i was drawn from i.i.d. $N(0,1)$, with $i = 1, \ldots, 94$ (as in the DeepSAGE data), whereas the gene expression levels were simulated as follows,

1 Considering a i.i.d. random effects, $\epsilon_i \sim N(0,1)$, the "connected" genes 1 and 2, were drawn from

$$\begin{aligned} Y_{i1} &\sim \text{Poisson}(\lambda_{i1}), \quad \log(\lambda_{i1}) = X_i + \epsilon_i \\ Y_{i2} &\sim \text{Poisson}(\lambda_{i2}), \quad \log(\lambda_{i2}) = \epsilon_i \end{aligned}$$

Note that only gene 1 is affected by the confounder X.

2 The genes 1 and 2 are "unconnected" and drawn from

$$\begin{aligned} Y_{i1} &\sim \text{Poisson}(\lambda_{i1}), \quad \log(\lambda_{i1}) = X_i \\ Y_{i2} &\sim \text{Poisson}(\lambda_{i2}), \quad \log(\lambda_{i2}) = 0 \end{aligned}$$

Note that, again, only gene 1 is affected by the confounder X.

3 Considering a i.i.d. random effects, $\epsilon_i \sim N(0,1)$, the "connected" genes 1 and 2, were drawn from

$$\begin{aligned} Y_{i1} &\sim \text{Poisson}(\lambda_{i1}), \quad \log(\lambda_{i1}) = X_i + \epsilon_i \\ Y_{i2} &\sim \text{Poisson}(\lambda_{i2}), \quad \log(\lambda_{i2}) = X_i + \epsilon_i \end{aligned}$$

Note that both genes 1 and 2 are affected by the confounder X.

4 The genes 1 and 2 are "unconnected" and drawn from

$$\begin{aligned} Y_{i1} &\sim \text{Poisson}(\lambda_{i1}), \quad \log(\lambda_{i1}) = X_i \\ Y_{i2} &\sim \text{Poisson}(\lambda_{i2}), \quad \log(\lambda_{i2}) = X_i \end{aligned}$$

Note that, again, both genes 1 and 2 are affected by the confounder X.

We performed 1000 iterations for each of the four scenarios and inferred the (partial) correlation coefficient between the genes. As can be seen from the results in Figure 1, the inferred values for the relation between genes 1 and 2 on average exactly correspond to the expected value, i.e., non-zero and zero two times.

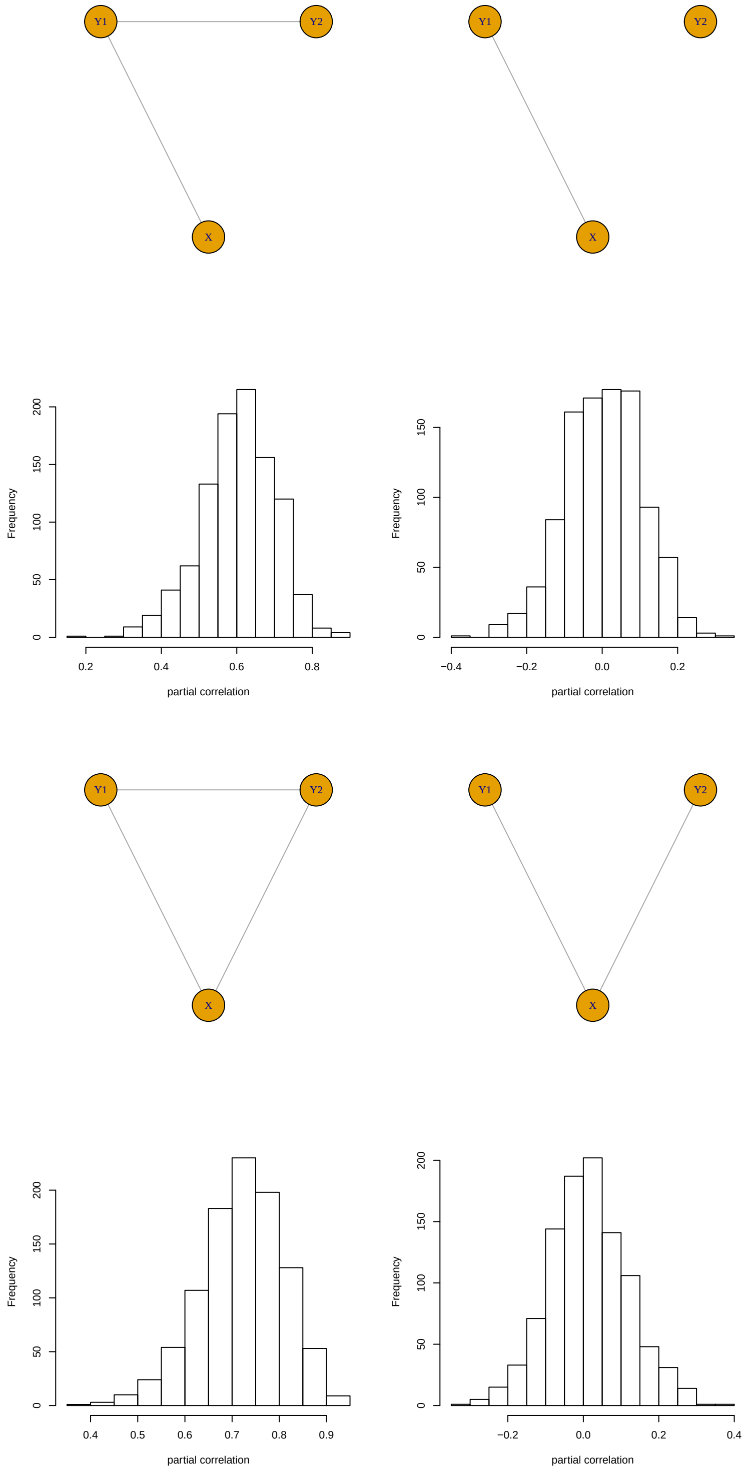

Figure 1 Inferred partial correlation coefficient by means of the two-step inference procedure (Poisson regression + Graphical Lasso) between genes 1 and 2 for the four different confounding plus interaction scenarios indicated by the networks above each of the corresponding histograms.
